# Evaluating the Validity and Reliability of the Gender Equitable Men’s Scale Using a Longitudinal Cohort of Adolescent Girls and Young Women in South Africa

**DOI:** 10.1007/s10461-021-03436-0

**Published:** 2021-08-24

**Authors:** Paul D. Wesson, Sheri A. Lippman, Torsten B. Neilands, Jennifer Ahern, Kathleen Kahn, Audrey Pettifor

**Affiliations:** 1 Department of Epidemiology and Biostatistics, University of California, San Francisco, 550 16th St., 3rd Floor, UCSF Box 0886, San Francisco, CA 94143 USA; 2 Center for AIDS Prevention Studies, University of California, San Francisco, 550 16th St., 3rd Floor, San Francisco, CA USA; 3 Division of Epidemiology and Biostatistics, School of Public Health, University of California, Berkeley, Berkeley, CA USA; 4 MRC/Wits Rural Public Health and Health Transitions Research Unit (Agincourt), School of Public Health, Faculty of Health Sciences, University of the Witwatersrand, Johannesburg, South Africa; 5 Gillings School of Global Public Health, University of North Carolina, Chapel Hill, USA

**Keywords:** Adolescent girls, HIV/AIDS, Gender norms, Item Response Theory, Measurement invariance

## Abstract

Inequitable gender norms and beliefs contribute to increased sexual risk behavior, and, among adolescent girls and young women (AGYW), risk of HIV acquisition. We investigated the longitudinal measurement properties of the Gender Equitable Men’s Scale (GEMS) when applied to a cohort of AGYW in rural South Africa (2011–2015). We used item response theory [Person-Item maps, Differential Item Functioning (DIF)] and measurement invariance confirmatory factor analysis models to assess the validity and reliability of the GEMS instrument. Item difficulty and endorsement of gender equitable beliefs both shifted over time. DIF analysis identified item bias for over half of the items; influenced by age, pregnancy, sexual debut, and intimate partner violence. Measurement invariance models revealed strong longitudinal invariance properties. GEMS is a reliable longitudinal measurement of gender equitable beliefs, with notable bias for specific items when administered to subgroups. Additional items specific to the adolescent experience are warranted for a more stable assessment of gender equitable beliefs in a population facing shifting norms as they mature.

## Introduction

Gender norms, defined as social expectations for appropriate behaviors for men and women [1], are a significant social determinant of health. Inequitable gender norms are associated with numerous health outcomes, including poor mental health (e.g. anxiety and depression), cardiovascular reactivity in response to stress, as well as asthma and musculoskeletal disorders due to occupational exposures [2–5]. Previous studies have also linked inequitable gender norms to risk of HIV acquisition; including perpetuation of intimate partner violence, alcohol abuse, and sexual concurrency among men [4, 6]. Relationship power dynamics, resulting from inequitable gender norms, that restrict female agency or promote male risk taking may facilitate the conditions that increase risk for HIV acquisition. Although associations are consistently found between gender norms and behavioral risks, the bulk of the evidence stems from studies that are not longitudinal and therefore cannot establish causation [7]. Studies designed to intervene on gender norms have shown promising short-term results with respect to intermediaries of HIV acquisition (e.g., improved negative attitudes towards women, increased condom use [8]) but the long-term effects of these interventions (e.g., HIV prevention) are less well-established because of short follow-up periods and lack of biological measures collected [9]. Collectively, these studies linking gender norms to risk of HIV acquisition contribute to the evidence base that support the U.S. President’s Emergency Plan for AIDS Relief (PEPFAR) and other international organizations prioritizing interventions focused on promoting gender equitable norms as a strategy to combat the global HIV epidemic [10–12].

Gender norms have been measured most commonly with the Gender Equitable Men’s Scale (GEMS). The scale, first developed by Pulerwitz and Barker to study gender norms among men in Brazil [1], has been implemented in many populations (including men and women) around the world to measure gender equity. Inequitable gender norms, as measured by GEMS, have been associated with multiple HIV-related outcomes, including sexual concurrency, intimate partner violence perpetration (by men), alcohol abuse, and HIV acquisition [6, 13]. Pulerwitz et al. recently used the GEMS instrument to investigate how gender norms influence HIV testing and treatment uptake in a survey of rural men and women in South Africa, finding that greater endorsement for inequitable gender norms was associated with decreased odds of current ART use among both men and women [14]. Similarly, Sileo et al. found that, among Ugandan fishermen living with HIV, endorsing more inequitable gender norms (assessed using the GEMS instrument) was associated with greater internalized HIV stigma, greater likelihood of missing HIV clinic appointments, and being less adherent to ARTs. Leveraging longitudinal data from a cohort of adolescent girls and young women (AGYW), we used the GEMS instrument to investigate the multi-level influences of gender norms on HIV and HSV-2 acquisition. We found that a more gender equitable social environment (i.e., gender equitable beliefs among one’s peers in school) was protective against HIV and HSV-2 acquisition [15]; and, as others have noted, that inequitable held beliefs were associated with intermediaries of HIV risk (e.g., unprotected sex, intimate partner violence, sexual concurrency), consistent with other studies [6, 15].

Previous validation studies of the GEMS instrument have used factor analysis techniques with cross-sectional samples to test the hypothesized structure of the underlying gender norms construct measured by the GEMS instrument, and to yield empirical evidence that individual items are sufficiently associated with each other and this underlying construct [16, 17]. These validation studies have also been used to adapt the GEMS instrument to local contexts and to provide evidence for restricting the instrument to a subset of items that optimally measure gender equity among a subgroup of the population (e.g. different items are optimal for adult males vs adult females). For example, Vu et al. recently evaluated the GEMS instrument in an adolescent population in Uganda, and confirmed that the scale was invariant by gender (males compared to females) but factor loadings differed for some items when comparing age groups (10–14 year olds compared to 15–24 year olds) [17]. This study, however, was limited by its cross-sectional design, which prevented the assessment of GEMS invariance across time as participants aged into adulthood. Additionally, the study by Vu et al. calculated the GEMS score by summing the number of items in which the participant disagreed with the gender inequitable statement. Such an approach, while common, weights each item equally and the final GEMS score is determined irrespective of which gender inequitable statements were endorsed. This approach offers limited insight into the dimensions of gender (in)equity that are endorsed by the adolescent population, and how experiences during adolescence may shape response patterns to the GEMS instrument. To the best of our knowledge there has not been a longitudinal validation of the GEMS scale; that is, an evaluation of the GEMS instrument’s consistent measurement of the same underlying construct within the same population over time.

Adolescence is a key developmental stage and transition point in the lifecourse. As young people transition into adulthood and explore their sexuality, they face a constellation of exposures that are either harmful or protective against their risk of HIV acquisition [18]. In South Africa, specifically, adolescence is a vulnerable period for girls and young women with respect to HIV acquisition and other sexually transmitted infections (STIs). In fact, adolescent girls in South Africa are over four times as likely to become infected with HIV compared to their male counterparts [19]. More broadly, adolescent girls in sub-Saharan Africa account for 75% of new HIV acquisitions among young people 15–19 years of age [20]. Given the high vulnerability to HIV acquisition for the adolescent population, and the evidence linking gender norms to HIV acquisition and current and future programming designed to modify norms, it is important to understand the measurement properties of the GEMS instrument for this population.

To fill this gap in the literature, we used a longitudinal cohort of AGYW in Mpumalanga Province, South Africa. Our objective was to evaluate the longitudinal validity and reliability of the GEMS instrument in a population experiencing developmental transitions.

## Methods

The HIV Prevention Trials Network (HPTN) 068 was a randomized controlled trial set in Mpumalanga Province, South Africa to study the effect of increasing adolescent girls’ school attendance on risk of HIV acquisition. Study participants were randomized to either receive a monthly cash transfer conditional on school attendance or no cash transfer. Potential participants were eligible to participate in HPTN 068 if they were enrolled in school grades 8–11, not married or pregnant, able to read, had the necessary documentation to open a bank account, had a parent or guardian with the necessary documentation to open a bank account, and (at the time of enrollment) resided in the study area and intended to remain until the completion of the trial. Full details of the RCT and primary results are described elsewhere [21].

Beginning in 2011, 2533 AGYW were enrolled in the study and followed for up to 5 years, with annual surveys measuring aspects of their social and economic life. During the main trial period, 2011 through 2015, participants were only eligible for the annual survey if they were currently in school (grades 8 through 12). Following the completion of the conditional cash transfer trial, the study team implemented a postintervention survey, which re-engaged participants who were no longer in secondary school. We restricted our longitudinal analysis to the first, third, and fifth years of the study, corresponding to the visits with the highest participant retention. Surveys were administered to study participants via an audio computer-assisted self-interview. Annual follow-ups also included HIV and STI testing. The GEMS instrument was part of the annual survey administered to study participants. The original GEMS instrument included 24 items. A previous analysis of the GEMS instrument found that, in adapting the instrument to the South African context, restricting the entire instrument to 13 items was optimal when measuring gender equitable beliefs among adult women [22]. We therefore restricted our analysis of the GEMS instrument to the 13 items that were previously determined to be a valid measurement of gender norms among women. All items were phrased in a gender inequitable way (e.g. *It is the man who decides what type of sex to have*). For each item, participants could respond (1) *Agree a lot*, (2) *Somewhat Agree*, or (3) *Do not agree at all*. Category Characteristic Curves from a prior analysis indicated no significant difference between the *Agree a lot* and *Somewhat Agree* categories; therefore, we collapsed these two responses into a single “Agree” response category [15]. Higher scores on the GEMS scale indicated greater disagreement with the gender inequitable statement, and therefore greater endorsement in gender equitable beliefs.

### Analysis

We used exploratory factor analysis to assess the number of factors that are measured using the GEMS instrument. Cronbach’s alpha was used to measure the internal consistency of the 13-item set at each study visit.

### Instrument Validity

We used several analytical techniques from Item Response Theory (IRT) to assess the measurement properties of the GEMS instrument. Item Response Modeling (IRM) is a popular tool in the education and psychometrics literature [23, 24]. In contrast to Classical Test Theory, which treats each item (or question) as equally difficult and calculates an overall score by summing the number of items answered “correctly”, IRT calculates individual scores (or level of proficiency) based on *which* questions were answered “correctly” rather than *how many* questions were answered “correctly”. In the case of the GEMS instrument, items are not inherently correct or incorrect, but are either endorsed norms or are not endorsed. For the purpose of our analysis, we scored items from the GEMS instrument as “correct” if participants responded, “Do not agree at all”, thereby indicating more equitable norms for that statement. The difficulty of an item, for a given population, is calculated based on how many (of those assessed) answered that item correctly. Individuals are also assigned a proficiency (or ability) score, based on which items they answered correctly. The item difficulty and the individual proficiency score, both measured in logits (defined as the natural log of the odds ratio), may be converted to probabilities to predict the likelihood that an individual would answer a specific item correctly [23]. We calculated the logit score for each item of the GEMS instrument at baseline and at the last visit. Here, we focused on the first and last visit to allow time for developmental changes and life experiences to occur so as to make the strongest possible comparison for the stability of GEMS item difficulty over time. Items were then rank ordered according to their estimated difficulty at baseline and the last visit, and rankings were compared across time. Person-Item maps were used to visualize the item difficulty ranking. Person-Item maps (also known as Wright Maps) plot the logit location of each item; items with lower logit scores are relatively easy items to endorse, whereas items with higher logit scores are more difficult to endorse. Person-Item maps also plot the distribution of logit scores for each individual respondent, visualizing the distribution of the level of proficiency in the latent construct that is being measured.

Differential Item Functioning (DIF) measures whether, on average, subgroups of respondents answer items differently (i.e., are more or less likely to endorse an item) according to the defining characteristic of the subgroup. We used DIF analysis to assess differential response by four characteristics defined at baseline: age (≤ 15 vs. ≥ 16; dichotomized based on the age distribution), pregnancy history (ever pregnant vs. never pregnant), sexual debut (never had sex vs. ever had sex), and intimate partner violence (IPV) history (never experienced IPV vs. ever experienced IPV).

### Instrument Reliability

Finally, we used confirmatory factor analysis (CFA) to test for measurement invariance. Measurement invariance refers to the reliability of an instrument administered to different groups or repeatedly measured within the same group [25]. Data are fit to a series of nested models with increasing parameter constraints. For our purposes, the parameter constraints force the equivalence of parameter values of the instrument when administered to the same population on different testing occasions. Goodness of fit statistics are used to assess model fit, including the Comparative Fit Index (CFI), the Standardized Root Mean Square Residual (SRMR), and the Root Mean Squared Error of Approximation (RMSEA). Models with a CFI estimated to be greater than 0.9 are considered to have an acceptable fit, whereas greater than 0.95 is considered a good fit [26]. Models with a SRMR value less than 0.08 are considered to have good fit [27]. Models with an RMSEA value between 0.05 and 0.08 are considered to have an acceptable fit, whereas RMSEA values between 0.01 and 0.05 are considered to have a close fit.[26]. When comparing nested models, a change in CFI of less than -0.004 (or a change of less than 0.01 in the RMSEA) indicates that adding more equality constraints does not substantially decrease model fit, and the more constrained model is not significantly worse than the prior model [26, 28]. We tested four levels of measurement invariance (with each level corresponding to increasing model constraints): (1) *Configural Invariance* (in which the same factor structure is represented at each time point), (2) *Strong Invariance* (in which factor loadings are constrained to be equal [signifying that the latent construct has the same meaning at each time point], and equal item intercepts [indicating that influences unrelated to the common factor do not systematically cause higher or lower item responses at each time point]), (3) *Scalar Invariance* (in which item intercepts, factor loadings, and construct means are constrained to be equal across time points), and (4) *Strict Invariance* (in which item residual variances, factor loadings, and intercepts are all constrained to be equal across time points).

Analyses were conducted using Stata version 16 and R statistical software version 3.6.1 [29, 30]. The R packages, “eRm” [31], “ltm” [32], and “difR”[33] were used to conduct the IRM analyses (Person-Item maps, DIF); “lavaan”[34] and “semTools”[35] were used to implement the measurement invariance analysis.

### Ethical Approval

Institutional review board approval for the HPTN 068 cohort study was obtained from both the University of North Carolina at Chapel Hill and the University of the Witwatersrand Human Research Ethics Committee. Ethical approval for this analysis was also obtained from the institutional review boards at the University of North Carolina-Chapel Hill, the University of California-San Francisco, the University of California-Berkeley, and the University of the Witwatersrand.

## Results

There were 2533 AGYW who enrolled in the HPTN 068 cohort at baseline (visit 1). Mean age of participants was 15.5 years at the baseline visit, and 20.2 years at the last visit. Most participants at each visit answered the GEMS questions and received a GEMS score (range 51.3–99.9%; Table 1). At baseline, 8.9% of respondents had ever been pregnant. This proportion rose to 37.8% of respondents at the last visit (visit 5). Similarly, the proportion of respondents who had ever had sexual intercourse rose from 27.4% at the baseline visit to 64.7% at the last visit. Just over 10% of the cohort had experienced IPV in the 12 months preceding the survey at the baseline visit. This proportion peaked at 23.5% at visit 3, when the majority of respondents were enrolled in grades 10 and 11, and declined to 9.5% by the last visit.

**Table 1:** Descriptive characteristics of the HIV Prevention Trials Network (HPTN) 068 cohort and performance of the Gender Equitable Men’s Scale at study visits 1, 3, and 5; Mpumalanga Province, South Africa

	Visit 1	Visit 3	Visit 5
n	2533	1870	2185
GEMS score (%)	2530 (99.9%)	960 (51.3%)	1937 (88.6%)
Age (mean [SD]) [Minimum, Maximum]	15.5 (1.66) [13, 21]	17.1 (1.49) [14, 22]	20.2 (1.45) [17, 26]
Have ever been pregnant	223 (8.9%)	329 (18.2%)	732 (37.8%)
Ever had sex	693 (27.4%)	638 (34.1%)	1,255 (64.7%)
Experienced intimate partner violence in the past 12 months	269 (10.9%)	434 (23.5%)	182 (9.5%)
Grade Enrolled			
Grade 8	640 (25.3%)	14 (0.8%)	0
Grade 9	682 (26.9%)	80 (4.4%)	0
Grade 10	699 (27.6%)	714 (39.6%)	12 (1.2%)
Grade 11	512 (20.2%)	661 (36.6%)	153 (14.9%)
Grade 12	0	335 (18.6%)	358 (34.8%)
University	0	0	506 (49.2%)
Cronbach’s alpha	0.775	0.841	0.851
Factor Loadings			
The man decides what type of sex to have	0.463	0.507	0.476
Men always ready to have sex	0.436	0.500	0.462
Men need sex more than women	0.391	0.478	0.456
A woman should not initiate sex	0.423	0.489	0.494
Woman who has premarital sex deserves no respect	0.401	0.424	0.472
Women who carry condoms are easy	0.382	0.459	0.534
Real women have children	0.492	0.569	0.591
A real man produces a male child	0.496	0.568	0.628
Childcare is a mother’s responsibility	0.498	0.626	0.636
Taking care of home/family woman’s role	0.484	0.618	0.596
Taking care of home/family woman’s role	0.440	0.552	0.597
Man should have final word re: home decisions	0.551	0.656	0.679
Woman should obey husband in all things	0.493	0.580	0.589

The Cronbach’s alpha for the GEMS score at each visit exceed the 0.7 threshold, indicating sufficient internal consistency. Factor loadings for each of the 13 GEMS items were generally low, most estimated to be between 0.3 and 0.6 at each time point. Although the factor loadings for each item exceeded the acceptable threshold, there was variability in the estimated factor loading for several items across visits, suggestive of individual items inconsistently measuring the underlying construct over time.

**Fig. 1:**
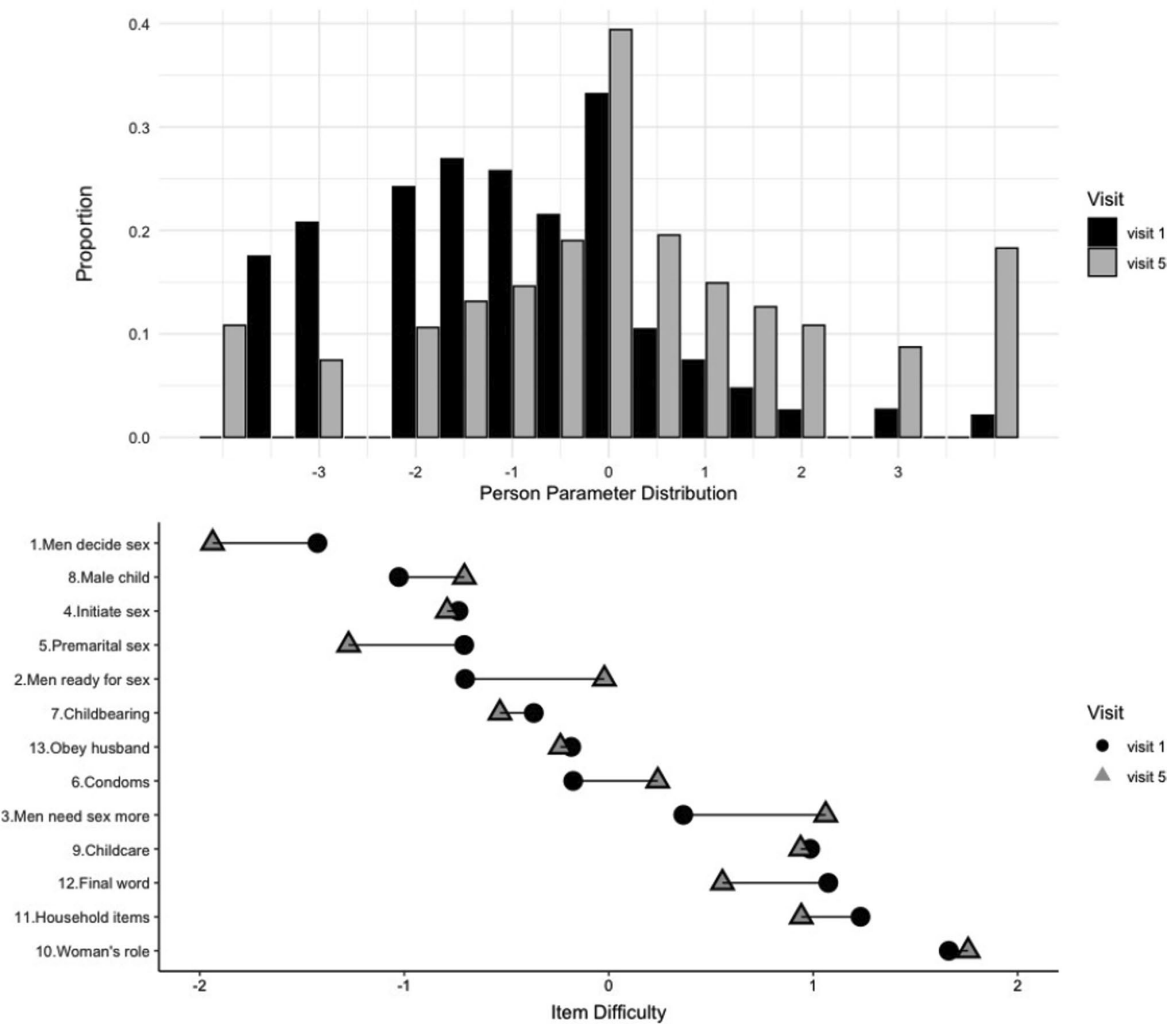
Person-Item map of Gender Equitable Men’s Scale (GEMS) at baseline (visit 1; n = 2533) and the last visit (visit 5; n = 2185); Mpumalanga Province, South Africa

The top panel of Fig. 1 shows the distribution of individual ability scores, comparing participants at baseline (visit 1) with participants at the last visit (visit 5). The distribution of participant abilities shifted over time, such that the study population at the last visit, five years after baseline, was more likely to make gender equitable responses than the same study population at baseline. The bottom panel of Fig. 1 shows the difficulty ranking (or the likehood of item endorsement) for each item at baseline and any change in item difficulty at the last visit. Most items showed substantial shifts in item difficulty over time (either becoming easier or harder over time). The following five items became harder to endorse (or agree with) over time: *The man decides what type of sex to have* (Item 1)*, A woman who has sex before she marries does not deserve respect* (Item 5)*, Only when a woman has a child is she a real woman* (Item 7)*, The husband should decide to buy the major household items* (Item 11)*,* and *A man should have the final word about decisions in his home* (Item 12). The following four items became easier to endorse (or agree with) over time: *A real man produces a male child* (Item 8)*, Men are always ready to have sex* (Item 2)*, Women who carry condoms on them are easy* (Item 6)*,* and *Men need sex more than women do* (Item 3). The following four items remained relatively unchanged in terms of difficulty to endorse over time: *A woman should not initiate sex* (Item 4)*, A woman should obey her husband in all things (Item 13), Changing diapers, giving a bath, and feeding kids are the mother’s responsibility* (Item 9)*,* and *A woman’s role is taking care of her home and family* (Item 10). Although there were shifts in individual item difficulty scores, item 1 (*It is the man who decides what type of sex to have*) and item 10 (*A woman’s role is taking care of her home and family*) remained the easiest to not agree with and the hardest to not agree with, respectively, at baseline and the last visit. For most other items, difficulty ranking changed from baseline to the last visit.

Seven of the 13 items showed evidence of differential functioning based on demographic characteristics or life experiences (p < 0.05) in the DIF analysis (Table 2). An additional two items were suggestive of DIF with p-values within the range of 0.05 and 0.1. Of the four demographic characteristics or life experiences, experiencing IPV coincided with the most items showing evidence for DIF (n = 6). For half of these items, the reference group (AGYW who had never experienced IPV) was more likely to not agree with the gender inequitable statement; for the other half of these items, the comparison group (AGYW who had ever experienced IPV) was more likely to not agree with the gender inequitable statement. One item, *A woman who has premarital sex deserves no respect*, showed evidence for DIF across all four categories of demographic characteristics and life experiences. For each of these categories, the group that was older or had experienced the life event was more likely to not agree with the gender inequitable statement. Only one other item showed evidence for DIF across the majority of categories. *A real man produces a male child*, showed evidence for DIF for all categories except for age. For each of these categories, those who had not experienced the life event were more likely to agree with the gender inequitable statement.

**Table 2:** Differential item functioning of the Gender Equitable Men’s Scale (GEMS) administered to adolescent girls and young women at the baseline visit (n = 2533); Mpumalanga Province, South Africa

Item	Age Reference = 15 and under Comparison = 16 and over			Pregnancy Reference = Never Pregnant Focal = Ever Pregnant			Sexual debut Reference = Never had sex Focal = Ever had sex			Intimate partner violence Reference = Never experienced Focal = Ever experienced		
	DIF	Group	p-value	DIF	Group	p-value	DIF	Group	p-value	DIF	Group	p-value
1. The man decides what type of sex to have							∙	Reference	0.049	∙	Reference	0.023
2. Men always ready to have sex	°	Reference	0.101							∙	Reference	0.021
3. Men need sex more than women				°	Comparison	0.066						
4. A woman should not initiate sex												
5. Woman who has premarital sex deserves no respect	∙	Comparison	0.005	∙	Comparison	0.006	∙	Comparison	< 0.005	∙	Comparison	< 0.005
6. Women who carry condoms are easy	°	Reference	0.080				°	Reference	0.073			
7. Real women have children				∙	Reference	0.028						
8. A real man produces a male child				∙	Reference	< 0.005	∙	Reference	< 0.005	∙	Reference	0.001
9. Childcare is a mother’s responsibility										∙	Comparison	0.011
10. Taking care of home/family woman’s role										∙	Comparison	0.021
11. Husband decides to buy major household items												
12. Man should have final word re: home decisions												
13. Woman should obey husband in all things												

∙ Flagged for DIF (p-value less than 0.05)

° Not flagged for DIF but marginal (p-value between 0.05 and 0.1)

“Group” refers to the group that is more likely to not endorse the statement

The results of the measurement invariance analysis showed that the Configural model, a single factor with all items loading onto that factor, fit the data well (CFI = 0.936, SRMR = 0.73, RMSEA = 0.04). Constraining the factor loadings, item intercepts, and thresholds to be equivalent across time did not substantially decrease the model fit (ΔCFI =  − 0.002, ΔSRMR = 0.00, ΔRMSEA = 0.000). However, constraining the factor means and the item residual variances to be equivalent over time did substantially decrease the model fit (Table 3). The “scalar invariance” model did not show acceptable goodness of fit statistics (that is, adding parameter constraints for the “scalar invariance” model did substantially reduce the model fit compared to the “strong invariance” model). Therefore, the analysis suggests that the GEMS instrument shows strong longitudinal invariance. Participant’s standings on the latent gender norms construct can and do change over time (i.e., AGWY are becoming more gender equitable over time), but the latent construct itself is not changing over time.

**Table 3:** Longitudinal measurement Invariance models for the Gender Equitable Men’s Scale (GEMS) administered to adolescent girls and young women at visit 1 (n = 2533), visit 3 (n = 1870), and visit 5 (n = 2185); Mpumalanga Province, South Africa

Model	Nested model comparison					Model fit indices			Differences in fit indices		
	DF	χ^2^	Δ χ^2^	Δ DF	p-value	CFI	SRMR	RMSEA	Ref model	Δ CFI	ΔRMSEA
(1) Configural	660	1509.0				0.936	0.073	0.040			
(2) Strong	682	1572.7	72.512	22	2.646e−07	0.934	0.073	0.040	1	− 0.002	0.000
(3) Scalar/Means	684	2228.0	282.825	2	< 2.2e−16	0.893	0.074	0.050	2	− 0.041	0.011
(4) Strict	710	2514.6	101.174	26	8.180e−11	0.894	0.076	0.049	3	0.001	− 0.001

RMSEA (exact fit = 0.00; close fit = 0.01–0.05; acceptable fit = 0.05–0.08; mediocre fit = 0.08–0.10; poor fit = greater than 0.10)

CFI (> 0.9 = acceptable fit; > 0.95 = good fit)

SRMR (< 0.08 = good fit)

Δ CFI <  − 0.004 = indicates adding more equality constraints did not substantially decrease model fit, the latter (more constrained) model is not significantly worse

ΔRMSEA < 0.010 = indicates adding more equality constraints did not substantially decrease model fit, the latter (more constrained) model is not significantly worse

*DF* Degrees of Freedom, *CFI* Comparative Fit Index, *SRMR* Standardized Root Mean Square Residual, *RMSEA* Root Mean Squared Error of Approximation, *Strong* simultaneously constrain factor loadings, thresholds, and intercepts (necessary to constrain all three because items are binary)

## Discussion

In this study, we evaluated the validity and reliability of the GEMS instrument in a longitudinal setting among a population in developmental transition from adolescence to young adulthood. Our findings are consistent with other studies in terms of demonstrating sufficient internal consistency and loading on a single construct [5]. We additionally have demonstrated that the GEMS instrument is a reliable measure of gender equitable beliefs over time, with measurement invariance analyses indicating that the GEMS instrument has strong longitudinal invariance properties; the same underlying construct is measured over time. However, the means of the underlying construct over time are not invariant; as participants age and experience more life events, this cohort became more gender equitable over time.

Although the GEMS instrument, as a whole, appears to be reliable over time, our results also raise concern for the performance (or validity) of individual items. The DIF analysis flagged nine of the 13 items as operating differently between the reference and comparison groups for at least one demographic or life experience category (p < 0.1). Item 5, *A woman who has sex before she marries does not deserve respect*, was flagged for DIF for all four categories. For each category, it was easier for the group that was older or who had experienced the life event to disagree with item 5. Many of the items flagged at the first visit for DIF, or item bias, directly related to one’s personal experiences. For example, AGYW who had never been pregnant at the baseline visit were less likely to agree with item 7 (*Only when a woman has a child is she a real woman*), while AGYW who had been pregnant prior to the baseline visit were less likely to agree with item 5 (*A woman who has sex before she marries does not deserve respect*). Experiencing IPV in the 12 months preceding the baseline survey impacted responses to six of the 13 items. Interestingly, of those six items, AGYW who had experienced IPV were less likely to agree with the three items that pertained to the woman’s traditional role in the household and to female sexuality. In contrast, AGYW who had never experienced IPV were less likely to agree with the remaining three items which pertained to male sexuality. Notably, three out of the four items that were not flagged for DIF by any of the life experience categories addressed household power dynamics and the authority of the husband over his wife. Since study eligibility included not being married at enrollment, these statements were more hypothetical for participants to respond to (drawing from their own observations and norms in the community); direct lived experiences did not inform participant responses. Alternatively, these items related to power may be more consistently understood and normative over the transition to adulthood.

Our results highlight the importance of evaluating an instrument holistically, as well as the individual items. While our analyses indicate that the GEMS instrument is a reliable assessment of gender equitable beliefs, results also provide evidence for item bias differentially impacting subgroups. As a direct implication, these results should caution researches against pulling individual items from an instrument to include in a separate survey and interpreting those individual items as a proxy for what the instrument as a whole is measuring. This practice could result in measurement error if items systematically perform differently based on lived experiences, or other demographic characteristics. With respect to GEMS specifically, the evidence for individual item bias tied to experiences during this transitional stage of the life-course may also warrant a separate set of items that are designed specifically for the adolescent experience [36, 37].

Additionally, our IRT analysis of the change in item difficulty ranking from the baseline assessment (visit 1) to the last assessment (visit 5) shows that the AGYW cohort, as a whole, became more gender equitable over time. This may reflect an age effect in a group going through maturation. Of course, age is likely not the sole factor responsible for the cohort becoming more gender equitable over time. Correlated with aging into adulthood, this cohort also acquired higher educational attainment, became sexually active, experienced a pregnancy, and experienced intimate partner violence by visit 5; all of which have been shown to be associated with endorsement of gender equity. Even after accounting for some of these life experiences, increasing age has been shown to be independently associated with greater endorsement of gender equity among young women in other settings [38]. Results from an overlapping community randomized controlled trial in the study area also suggest that background gender norms in the community were improving irrespective of the community intervention, which included an equitable gender norms component [39]. The overall shift in community endorsement of gender equity is likely due to increased media exposure through increased access to TV programming and smartphones in the study area. In this setting, these period effects may be impossible to disentangle from age effects and the effect of life experiences on endorsing gender equity. Collectively, these results should caution investigators against interpreting improvements in GEMS scores alone as a reflection on the success of an intervention designed to improve gender norms in younger age groups [40, 41].

This analysis includes several limitations. Although analyses accounted for repeated measurements over time within the same individual, our software tools could not account for additional clustering of data by village (girls came from 30 villages). It is possible that respondents who are clustered in the same school or the same village are more alike in their responses than respondents who come from different schools and villages. If this were the case, not accounting for clustering could bias our results towards the null and reduce statistical power, limiting our ability to detect significant differences in responses. Additionally, we restricted our analysis of the original 24-item set to the 13 items previously established as the ideal subset when administering the GEMS instrument to adult females. While our reliability results are consistent with previous validation studies of the GEMS instrument, our IRM and measurement invariance results may be impacted when using the full 24-item instrument in this population.

This is the first examination of measurement properties of the widely used GEMS instrument in a longitudinal cohort. Our findings indicate that, while holistically the instrument is a reliable measure of gender equitable beliefs, a number of individual items are differentially influenced by lived experiences. When applying the GEMS instrument to any population, but particularly among youth, investigators should perform a DIF analysis to assess the validity of the instrument in that population. We also recommend that future applications of the GEMS instrument in the adolescent population include items relevant to this transitional period that are more stable in the face of shifting norms.

## Data Availability

Data access is managed by FHI360 and data requests can be made by contacting Erica Hamilton at EHamilton@fhi360.org.

## Code availability

Code for statistical analysis is available upon request.
